# Artificial Organelles: Digital Microfluidic Platform for Proteoglycan and Glycoprotein Biosynthesis

**DOI:** 10.1100/tsw.2010.95

**Published:** 2010-06-01

**Authors:** Jeffrey G. Martin, Julie M. Beaudet, Jonathan S. Dordick, Robert J. Linhardt

**Affiliations:** ^1^ Department of Chemistry and Chemical Biology, Rensselaer Polytechnic Institute, Center for Biotechnology and Interdisciplinary Studies, Troy, NY, USA; ^2^ Department of Biology, Rensselaer Polytechnic Institute, Center for Biotechnology and Interdisciplinary Studies, Troy, NY, USA; ^3^ Department of Chemical and Biological Engineering, Rensselaer Polytechnic Institute, Center for Biotechnology and Interdisciplinary Studies, Troy, NY, USA

**Keywords:** artificial organelle, Golgi, endoplasmic reticulum, proteoglycan, glycoprotein, glycosaminoglycan, heparin, heparan sulfate, digital microfluidics

## Abstract

The development of artificial organelles is a new, fast-growing field in which a variety of techniques have been employed. This article gives a brief overview of the history of artificial organelles, and describes the development of an artificial endoplasmic reticulum and Golgi organelle on a digital microfluidic platform. This device should be useful in high-throughput combinatorial proteoglycan/glycoprotein synthesis, providing critical information about the control of biosynthetic pathways for these important signaling molecules.

Jeffrey G. Martin[1,2], Julie M. Beaudet[1,2], Jonathan S. Dordick[2,3], and Robert J. Linhardt[1,2,3,*]

The Golgi organelle, discovered in 1898 by Camillo Golgi, is responsible for the post-translational modification of proteins secreted from the endoplasmic reticulum (ER) in eukaryotic cells (Fig. 1a)[1]. Glycosylation, the most prominent post-translational modification, results in the biosynthesis of proteoglycans (PGs) and glycoproteins[2]. Glycosylation is not template driven, and it is hypothesized that the process is regulated by the unique substrate specificities and sequential action of the biosynthetic enzymes involved and by the Golgi compartmental structures housing the biosynthetic enzymes. The average mammalian Golgi consists of ~100–150 membrane-bound stacks termed cisternae, which hold hundreds of different Golgi enzymes[3]. Inside the cisterna, proteins are glycosylated by Golgi enzymes to produce biologically active PGs. The glycosaminoglycan (GAG) chains of PGs are among the most structurally complex and biologically important glycans[4]. The GAG structure, particularly heparan sulfate (HS), carries immense amounts of biological information[5]. By binding to biologically important proteins, GAGs play roles in a wide array of physiological processes, including blood coagulation, cell-cell and cell-matrix interactions, lipid transport and clearance/metabolism, host defense and viral infection mechanisms, and inflammatory processes[6]. Due to the physiological importance of PGs, it is imperative to learn more about their production and how the intricate process of glycosylation is regulated. Unfortunately, the complexity of the Golgi apparatus limits our understanding of HS biosynthesis. Because studying the *in vitro* enzymatic synthesis of HS in the absence of a Golgi does not afford full comprehension of the control and regulation of HS biosynthesis, we undertook the design and fabrication of an artificial Golgi using a digital microfluidic platform (Fig. 1b) that conceptually resembles the natural organelle[7,8].

The primary goals of the fabrication and operation of artificial organelles are to create reaction vessels for the construction of biologically significant products, and to employ such a platform to gain a fundamental understanding of organelle structure and function. Many current examples of artificial organelles consist of polymeric shells that surround aqueous medium[9,10,11,12,13]. Researchers have used channel microfluidics to produce cell-sized lipid vesicles[14]. These vesicles were shown to serve as

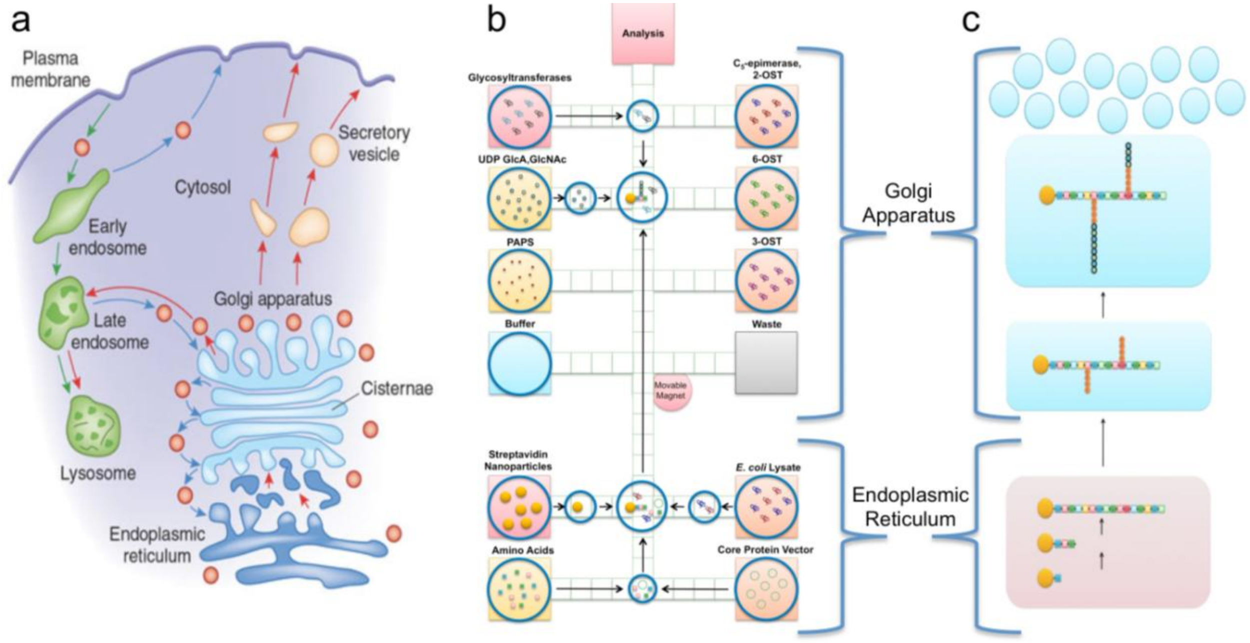

**FIGURE 1**. An artificial Golgi and artificial ER are inspired from their natural counterparts. (a) A cartoon depicting the Golgi and ER of a eukaryotic cell. Proteins are synthesized in the ER, and then sent to the Golgi for post-translational modification resulting in glycoprotein and PG products. Modified from Xu et al.[8]. (b) The design of an artificial Golgi/ER digital microfluidic chip for the biosynthesis of the HS-PG is shown. The HS-PG core protein is first synthesized on a magnetic nanoparticle in the artificial ER by *in vitro* translation. The particle holding the core protein is then transferred into the artificial Golgi portion of the digital microfluidic chip where it is glycosylated to yield HS-PG. The large boxes (multicolor) are reagents and enzyme reservoir electrodes, and the small boxes (light green) are electrodes for droplet movement, mixing, and sequestration. Modified from Martin et al.[7]. (c) A cartoon depicting the process of *in vitro* translation and glycosylation in the artificial ER/Golgi device.

effective bioreactors in the cell-free expression of green fluorescent protein. Vesicles have also been used as bioreactors for the synthesis of ATP, and have been modified to include channel and surface proteins before carrying out enzymatic reactions[15,16,17,18,19,20,21]. Although these examples of bioreactors proved successful, they are all considered “one-pot” reactions in which all reactants are present in the reactor vessel at any given time. A means to control the sequential combination of reactants is necessary to better mimic the Golgi and ultimately understand the process of glycosylation in the organelle. Along these lines, we developed an artificial Golgi that utilizes droplet-based digital microfluidics rather than a polymer casing to contain and control reactions. Previously, digital microfluidics had been used for analytical measurements, including enzyme-based assays (e.g., glucose quantification), matrix-assisted laser desorption/ionization mass spectrometry sample preparation, polymerase chain reaction to amplify DNA, and cell-based assays[22,23,24,25,26].

Digital microfluidics facilitates open droplet movement across a 2-D grid-like surface by the process of electrowetting[27]. Such technology has opened up new opportunities to design nanoscale, biomimetic, droplet vesicle–based reaction systems. These systems can be used to mimic the natural fluid vesicles that operate in natural organelles such as the Golgi apparatus. Using digital microfluidics, recombinant enzyme technology, and magnetic nanoparticles, we have created a functional prototype of an artificial Golgi organelle[7,8]. This artificial Golgi enzymatically modifies GAGs immobilized onto magnetic nanoparticles, analogous with the function of the natural Golgi, which is responsible for the enzymatic modification of GAGs attached to proteins.

Our specific approach involved the enzymatic sulfation of HS GAG chains immobilized onto 600-nm magnetic nanoparticles. Sulfo groups were transferred from adenosine 3’-phosphate 5’-phosphosulfate (PAPS) to the 3-hydroxyl group of the D-glucosamine residue in an immobilized HS chain using D-glucosaminyl 3-*O*-sulfotransferase. After modification, the nanoparticles with immobilized HS exhibited increased affinity for their fluorescently labeled antithrombin ligand, as detected by confocal microscopy. Since the biosynthesis of HS involves an array of specialized glycosyl transferases, epimerases, and sulfotransferases, this approach should mimic the synthesis of HS *in vivo*.

The creation of this artificial Golgi prototype results from the confluence of artificial organelle development, bioenzymatic GAG synthesis, and digital microfluidic technology. Additional GAG bioenzymatic syntheses using the artificial Golgi are under way in order to learn more about the specificities of the Golgi enzymes and to construct microscale GAG libraries. This artificial Golgi is also being coupled to online liquid chromatography mass spectrometry (LC/MS) for the compositional analysis of the bioenzymatically synthesized GAG products.

The continuing development of artificial organelles is now focused on the design of an artificial ER based on a similar digital microfluidic platform (Fig. 1b). The artificial ER is being used for microscale *in vitro* synthesis of the PG protein core. The artificial ER will then be integrated with the artificial Golgi to carry out the complete synthesis of PGs (Fig. 1c). Varying the use of different enzyme isoforms, different reaction times, and different concentrations of enzymes and cofactors during the biosynthesis will afford PG/GAG libraries. The resulting biosynthetically defined PG/GAG libraries will then be sequenced and assayed for biological activities. This artificial ER/Golgi device will thus be used to study PG biosynthesis, GAG sequencing, library generation, and will also be applied to the synthesis of a wide variety of glycoproteins.

## ACKNOWLEDGMENTS

The authors acknowledge generous support from the NSF (0709099 and IIS0713517) and from the NIH (M090257).
